# A hundred years of basic science in medical education

**DOI:** 10.1007/s40037-016-0269-1

**Published:** 2016-05-20

**Authors:** Matt Sibbald, Alan Neville

**Affiliations:** McMaster University, Hamilton, Ontario Canada

For a century, we have been trying to integrate basic science into medical education, beginning with the Flexner report. Flexner, a high school teacher, an outsider to the practice of medicine and medical education, was commissioned to re-evaluate the medical education system [1]. He was chosen for his expertise as an educator, having critiqued the American schooling system and having explored pedagogical techniques. The for-profit, disjointed, non-standardized North American system was losing the respect of the public. Flexner laid the groundwork for a marriage between professors of university science and physician trainers in clinical arts. Flexner’s suggested curriculum with two years of laboratory-based science followed by clinical training was modelled after a system already in place at Hopkins, itself an import from Germany credited to William Welch. The revolution of medical education, spurred by the Flexner report, instilled scientific rigour into medical education curricula. Instruction by physician scientists even became a quality indicator [1]. This transition of medical education from the for-profit sector to the university setting was accomplished through the integration of basic science. Basic science was a tool to gain public trust through academic rigour. Basic science became an aid to protect and preserve professional autonomy.

The inclusion of basic science, and the new breed of academic physician scientists, minted to fulfil these new university roles, was not without critique. The famous William Osler feared that this new brand of clinician scientists would be too far removed from the lives of patients to understand the complexities of clinical care. Dr Duffy, haematologist Professor Emeritus at Yale, talks about the profession “losing its soul at the same time its body is clothed in a luminous garment of scientific knowledge” [1].

Furthermore, how clinicians were able to use this basic science information was called into question. Exploration into problem solving by clinicians revealed a surprising lack of transfer, an inability to apply basic science to clinical contexts [2]. This challenged the teaching of basic science in isolation. Perhaps because of these concerns, or because of disgruntlement with the large amounts of passive memorization of basic science, new methods of integrating basic science into the curriculum were introduced. Born at McMaster in 1969, problem-based learning was designed with the purpose of nesting basic science training within clinical problems to make basic science more usable [3, 4]. This approach was intended to develop scientific thinking, and facilitate retention and transfer of basic science knowledge.

An alternate approach was pioneered at the University of Calgary in 1990, where the medical curriculum was structured around schemas, or organizational charts that highlighted key variables of common clinical presentations or biologic processes [3]. This focused learners on a decision-making process rather than on basic science or clinical conditions. The design of a schema highlights small numbers of key variables, intended to help learners categorize and distinguish, a valued ability for trainees destined to become diagnosticians.

Both the McMaster and University of Calgary curricular reforms resulted in practical benefits. Both were tied to shorter medical curricula of three rather than four years, perhaps because discipline-focused courses could be replaced with a smaller number of blocks or units organized around body systems or functions. Despite these curricula being in place for many decades, data on these interventions were scarce, and showed disappointingly modest impact on transfer and retention [5, 6]. As a result, the preoccupation with the role of basic science in the curriculum remains.

In 2011, an environmental scan to identify priorities for medical education curricula highlighted the integration and timing of basic and clinical science education [7]. However, interviews with key stakeholders within the medical education community identified even more fundamental questions [8]. What do we consider a basic science? Where do the social sciences fit? Are communication skills any less relevant than anatomy? Bandiera et al. [8] suggested shifting the focus away from specific basic science disciplines to consider all foundational knowledge that can be applied to the practice of medicine.

In this issue of Perspectives in Medical Education, Lisk et al. investigate why integrating basic science may be of value [9]. They help develop the literature base on how and when integration of basic science improves knowledge retention. They studied musculoskeletal conditions, providing anatomy and causal mechanism information to one group of students, while providing control students with information on epidemiology and treatment. They found a large effect of this basic science anatomic information on diagnostic accuracy. In an attempt to better demonstrate the mechanism behind this effect, they employed a ‘diagnostic justification test’, where students were given the diagnosis and asked to explain why the diagnosis was correct. The group taught with basic science were more likely to identify a key diagnostic feature, one that distinguished the disease from similar diagnoses, rather than just a correct feature. This suggests that if diagnostic skills are the goal, then basic science might help learners deduce distinguishing features, in contrast to the explicit emphasis of these distinguishing features in schemas.

While it is tempting to conclude that basic science should be routinely integrated into teaching, it is worth carefully examining the materials used. The authors studied musculoskeletal conditions, which could be perceived as anatomic problems, supplementing the instruction with basic anatomy teaching. Whether or not teaching other basic sciences with less direct links to these disease states would have the same effect is open to debate. Would teaching musculoskeletal histology or the physiology of injury repair lead to a similar benefit in diagnostic accuracy? I suspect not. The tight linkages between anatomy and disease states in this study may overstate the benefit for conditions without such clear causal linkages between basic and clinical sciences. These benefits are most apt to be found in content where basic science helps learners construct causal pathways, a phenomenon thought to facilitate retention [10].

This is not to suggest that the teaching of basic science should be restricted only to where it facilitates clinical knowledge acquisition or decision-making. The original rationale for teaching science in medical school curricula still applies. It is worth remembering that basic science did not enter medical curriculum as an educational tool for transfer and retention, but as a tool to advance medical practice, ensure academic rigour, and preserve the professionalization of medicine.
